# The prevalence of germline pathogenic variants in Estonian colorectal cancer patients: results from routine clinical setting 2016–2021

**DOI:** 10.3389/fgene.2022.1020543

**Published:** 2022-11-08

**Authors:** Laura Roht, Mikk Tooming, Kadri Rekker, Hanno Roomere, Kadri Toome, Ülle Murumets, Ustina Šamarina, Katrin Õunap, Tiina Kahre

**Affiliations:** ^1^ Department of Clinical Genetics, Institute of Clinical Medicine, University of Tartu, Tartu, Estonia; ^2^ Department of Clinical Genetics, Genetics and Personalized Medicine Clinic, Tartu University Hospital, Tartu, Estonia; ^3^ Department of Laboratory Genetics, Genetics and Personalized Medicine Clinic, Tartu University Hospital, Tartu, Estonia

**Keywords:** colorectal cancer, hereditary cancer syndromes, routine clinical setting diagnostics, NGS, molecular genetics of CRC

## Abstract

**Background:** Colorectal cancer (CRC) is the third most common cancer in Estonia in both women and men. According to the Estonian National Institute for Health Development, in 2017, there were 357 new colon cancer only cases in women and 282 in men. For colorectal cancer, the number for men and women altogether was 1040 in the same year. In 2018, there were over 1.8 million new cases worldwide. The Mayo Clinic found in a prospective, two-year multi-site study of CRC patients that 15.5% of patients carried pathogenic germline variants (PGV), using an >80 gene Next Generation Sequencing (NGS) panel.

**Material and methods:** This retrospective study aimed to analyse the estimated prevalence of pathogenic/likely pathogenic germline variants in Estonian colorectal cancer patients using NGS in a routine clinical setting. We gathered five-year data (July 2016-July 2021) of colorectal cancer patients (mostly not selected for age or family history) tested with either Illumina TruSight Cancer (94 genes) or TruSight Hereditary Cancer (113 genes) NGS panels.

**Results:** Three hundred and fourteen NGS analyses were performed due to either CRC or polyposis in anamnesis and/or family anamnesis, including 126 CRC cases and 44 colorectal polyposis cases, while 144 were either healthy family members or had other types of cancers. While a known disease-causing variant was identified in 16.4% of all cancer patients tested, we found that 21.4% of CRC patients had such a variant. Among the 44 colorectal polyps cases *MLH1,* gene was the most affected one (25%), the second and third most affected genes were *MSH2* and *CHEK2*. Other genes with disease-causing variants found in CRC patients included *APC, BLM, BMPR1A, BRCA1, FANCM, MSH6, MUTYH, PMS2, SMAD4, SPINK1* and *VHL.*

**Conclusion:** Our result give an overview of genetic testing of CRC patients, the prevalence of disease-causing variants and their landscape in Estonia. According to Estonian data, only 2.7–6.1% of CRC patients are genetically tested, which is around ten times less frequently than breast cancer patients and their family members. The diagnostic yield of CRC patients is 21.4%, suggesting that genetic testing will likely improve timely diagnosis and outcomes.

## Introduction

According to the World Health Organization cancer is the second leading cause of morbidity and death in Europe. It is estimated that approximately 5–10% of all cancers are hereditary. Colorectal cancer (CRC) is the third most common cancer in Estonia for both women and men (www.tai.ee). Lynch syndrome, also called hereditary non-polyposis colorectal cancer, is the most frequent genetic cause of colorectal cancer, accounting for ∼3–4% of all colorectal cancers in the world (Sinicrope, 2018). Lynch syndrome is caused by disease-causing variants in mismatch repair (MMR) genes. Previously, we have estimated the prevalence of Lynch syndrome associated MMR gene variants to be 1:485 in the general population based on Estonian Genome Centre data (Roht et al., 2020). This frequency is lower than the reported prevalence of disease-causing variants in MMR genes in the world population, which was estimated to be 1:100–1:180 in a 2020 study (Cerretelli et al., 2020), and estimated at 1:279 in another study (Win et al., 2017). For all cancer patients, it has been suggested that among unselected patients with cancer, approximately 1 in 8 patients (or 13.3%) harbours a pathogenic gene variant (PGV) (Samadder et al., 2021a). Like other centres, we mostly follow the National Comprehensive Cancer Network (NCCN) guidelines (www.NCCN.org). According to American studies around half of CRC patients with genetic variants would not have been found if standard recommendations and guidelines had been followed (Samadder et al., 2021a; Samadder et al., 2021b). As personalized medicine expands year by year, including both sequencing based diagnostics and treatment options directed by molecular genetic findings, the standard criteria are now becoming obsolete, and the urgency to share knowledge and molecular findings worldwide is growing.

The primary goal of our study was to estimate the prevalence of pathogenic/likely pathogenic germline variants (PGV or LPGV) in colorectal cancer patients using next generation sequencing (NGS) in a routine clinical setting. The second aim was to describe the genetic landscape of Estonian CRC, and to compare this to frequencies and distributions of PGV/LPGV worldwide. The third aim of this study was to estimate the diagnostic yield of NGS in a routine clinical setting of CRC patients.

## Materials and methods

### Study group

We retrospectively reviewed the clinical and laboratory data of all CRC and polyposis cases sent for genetic testing between July 2016 to July 2021 at the Genetics and Personalized Medicine Clinic of Tartu University Hospital in Estonia. This is the sole genetics centre in Estonia and almost all molecular diagnostics in the country is performed there. CRC was defined as C18-C21 codes from International Classification of Diseases 11^th^ Revision (ICD-11). We defined polyposis as the development of numerous polyps (growths that protrude from a mucous membrane), and excluded cases of solitary polyps if genetic findings did not identify a known polyposis syndrome and/or if the family history was negative for CRC or polyposis.

**FIGURE 1:**
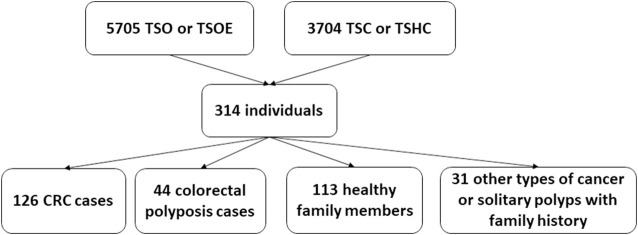
Overview of the study group.

During the five-year study period, 5705 Illumina TruSight One (TSO) and Illumina TruSight One Expanded (TSOE) and 3704 Cancer panel analyses were done. Three hundred fourteen NGS analyses were carried out because of CRC or polyposis in anamnesis and/or family anamnesis; of these, 126 individuals had CRC and 44 had colorectal polyposis. The remaining 144 were either healthy family members (113 individuals) or had other types of cancer or solitary polyps with family history (31 individuals) (Figure 1).

### Methods

Individuals in the cohort were sequenced using TruSight Cancer panel (TSC, 94 genes, Illumina Inc., San Diego, California) from 2015 until mid-2020, and with TruSight Hereditary Cancer panel (TSHC, 113 genes, Illumina Inc., San Diego, California) since mid-2020. For CRC relevant genes, it is notable that the TSHC panel includes *POLE* and *POLD1* genes which are absent from the TSC panel. Detection of copy number variation (CNV) of *BRCA1, BRCA2, TP53, CHEK2* and *CDH1* genes was carried out using DECoN (Fowler et al., 2016). In a few cases, where a hereditary cancer syndrome was suspected, but the gene was not covered on TruSight Cancer panels, or there was no finding, we also used TruSight One (TSO) and TruSight One Expanded (TSOE) panels (Illumina Inc., San Diego, California). These platforms cover ∼4800 and ∼6700 genes respectively, and are associated with known genetic disorders or clinical phenotypes. CNV analysis performed for TSO and TSOE panels for genes *BRCA1*, *BRCA2, TP53, CHEK2,* and *CDH1,* was carried out using CoNIFER (Krumm, 2012). We also report incidental findings in our routine setting based on criteria published by American College of Medical Genetics (ACMG), if the patient has given his/her consent (Miller et al., 2021).

All TruSight panels were sequenced on Illumina MiniSeq or NextSeq platform in a clinical setting. Raw sequencing reads were aligned to the hg19 reference genome using BWA MEM alignment algorithm, and variant calling was performed by Genome Analysis Toolkit (GATK) (McKenna et al., 2010). Variants were classified according to the criteria published by ACMG (Richards et al., 2015). The clinical relevance of all genetic variants was assessed using ClinVar (Landrum et al., 2018), InSight (https://www.insight-database.org/) and HGMD Pro databases, as well as the Genome Aggregation Database, gnomAD (Stenson et al., 2003; Karczewski et al., 2020). The pathogenicity of previously undescribed findings was evaluated using Varsome Clinical platform and/or other *in silico* protein functionality prediction programs (Kopanos et al., 2019). Copy number variants were detected for NGS data using either CoNIFER (v0.2.2) or DECoN (v1.0.2) software, or using the MLPA method. The following genes were tested by MLPA: *MLH1, MSH2, EPCAM, MSH6, MUTYH, PMS2, APC*, using their respective MRC HOLLAND (Netherlands) MLPA® kits: Probemix P003 MLH1/MSH2; Probemix P072 MSH6-MUTYH; Probemix P008 PMS2; and Probemix P043 APC.

Sanger sequencing was used to test family members when the disease-causing variant had already been detected.

### Statement of ethics

The study was approved by the Research Ethics Committee of the University of Tartu (274T-5, 14.11.2017 and 292/M-13, 15.04.2019).

## Results

**FIGURE 2:**
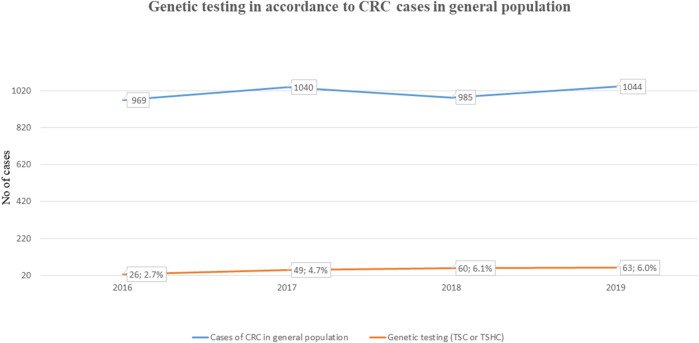
Fraction of individuals NGS tested for CRC (ICD-11 C18-C21) among total cases in general population.

Our retrospective study included 314 analyses carried out due to CRC or polyposis in anamnesis and/or family anamnesis. 55 PGV/LPGV variant positive individuals were identified amongst those screened using any of the platforms over the five-year study period (Table 1). One hundred seventy CRC and polyposis cases were investigated during the five-year study period, and the remaining 144 cases were either healthy family members, patients with non-CRC cancers, or individuals with solitary polyps with a relevant family history. Pathogenic or likely pathogenic germline variants (PGV/LPGV) were found in 38 out of 170 patients, which constitutes 22.3% of CRC and polyposis cases. As shown in Figure 2, only 2.7–6.1%, depending on the year, of CRC cases are genetically tested in Estonia. While this number is on the rise, Estonian CRC cases are still tested genetically for PGV approximately ten times less often than, e.g., breast cancer patients and their family members.

**TABLE 1:** Diagnostic yield of disease-causing variants in different clinical groups and healthy family members.

Group studied	No of findings	Diagnostic yield (%)
CRC cases (126)	27	21.4
Polyposis cases (44)	11	25.0
Healthy family members (113)	12	10.6
Other cases (other type of cancer or solitary polyps with family anamnesis (31)	5	16.1

Of all cases tested in our cohort, there were 27 cases of CRC in which a LPGV/PGV was identified, and 11 polyposis cases with LPGV/PGV identified. Altogether, we detected 12 LPGV/PGV in healthy family members (Table 1). Among the known or classic CRC related genes (*APC, AXIN2, BMPR1A, CHEK2, EPCAM, GREM1, MLH1, MSH2, MSH3, MSH6, MUTYH, NTHL1, PMS2, POLD1, POLE, PTEN, RPS20, SMAD4, STK11* or *TP53* gene) mismatch repair (MMR) genes disease-causing variants were the most prevalent (16/20) in our cohort, accounting for 80.0% of all cases. A LPGV/PGV in the *MLH1* gene was identified in one-third of cases (Table 2). We identified several cases of CRC or polyposis in which non-classical CRC genes were affected. These included two cases of *BRCA1* disease-causing variants, and additional variants in the genes *SPINK1, VHL, FANCM*, *BLM*, *APC*, *MUTYH, STK11, BMPR1A,* and *DSPP*. Further details about these cases are provided in supplementary materials.

**TABLE 2:** PGV or LPGV findings in typical and/or potential CRC- associated genes.

Gene	Variant	Clinical significance	Previously described	No of patients
MLH1	NM_000249.3 (MLH1):c.1976G>C, p. (Arg659Pro)	Pathogenic	Yes	3
MLH1	NM_000249.3 (MLH1):c.1918C>T, p. (Pro640Ser)	Likely pathogenic	Yes	1
MLH1	NM_000249.3 (MLH1):c.1668-1G>T, p.?	Likely pathogenic	Yes	1
MLH1	NM_000249.3 (MLH1):c.146T>A, p. (Val49Glu)	Pathogenic	Yes	1
MLH1	NM_000249.3 (MLH1):c.92C>A, (p.Ala31Asp)	Likely pathogenic	No (this study)	1
MLH1	NM_000249.3 (MLH1):c.55A>T, p. (Ile19Phe)	Pathogenic	Yes	1
MSH2	NM_000251.2 (MSH2):c.1164_1165delinsGT, p. (Asn388_Arg389delinsLys*)	Pathogenic	No (this study)	1
MSH2	NM_000251.2 (MSH2):c.1283_1284del, p. (His428Profs*14)	Pathogenic	No (this study)	1
MSH2	NM_000251.2 (MSH2):c.1661+5G>A, p.?	Likely pathogenic	No (this study)	1
MSH2	NM_000251.2 (MSH2):c.793-1G>A, p.?	Pathogenic	Yes	1
MSH2#	MSH2 exon 1–3 deletion and EPCAM exon 9 deletion	Pathogenic	Yes	1
MSH2#	MSH2 exon 1–6 and EPCAM exon 8–9 deletion	Pathogenic	Yes	1
CHEK2	NM_007194.4(CHEK2):c.319+2T>A, p.?	Likely pathogenic	Yes	2
CHEK2	NM_007194.4(CHEK2):c.1189del, p. (Val397Phefs*17) mosaic (12%)	Likely pathogenic	No (this study)	1
CHEK2	NM_007194.4(CHEK2):c.908+1540_1095+330del, p. (Met304Leufs*16)	Pathogenic	Yes	1
MSH6	NM_000179.2 (MSH6):c.3725G>A, p. (Arg1242His)	Pathogenic	Yes	1
MSH6	NM_000179.2 (MSH6):c.3514dup, p. (Arg1172Lysfs*5)	Pathogenic	Yes	1

^#^ legacy name of the deletion involving two genes (*MSH2* and *EPCAM*)

In our CRC patients, MMR associated PGV or LPGVs were the most common, making up 37.2% (16/43) of all the findings. Among MMR genes, *MLH1* was the gene most frequently affected, with variants identified in around 50% of MMR cases and 8/43 (18.6%) of all the cases with any genetic finding. The next most common gene with CRC-disease causing variants was *CHEK2*, with variants detected in five cases (11.6%). We identified three novel disease-causing gene variants in *MSH2* and one in both *MLH1* and *CHEK2* (Table 2) genes. In other common or potential CRC genes, including *APC, BLM, BMPR1A, BRCA1, FANCM, MSH6, MUTYH, PMS2, SMAD4, SPINK1* and *VHL,* novel variants were not found.

As described in the methods, several different methods were employed during five-year study period. In our cohort, the diagnostic yield for TSC was 33/238 (13.9%) and for TSHC 8/66 (12.1%). We did not find any disease-causing alterations in genes found exclusively in the TSHC panel, for example *POLE* and *POLD1* genes. The combined diagnostic yield for TruSight Cancer and TruSight Hereditary Cancer gene panels was 13.4%. (Details provided in Table 3.). On rare occasions, a larger panel than TruSight Cancer (such as TSO) was used in a clinical setting for cancer diagnostics; TSO diagnostic yield was 2/10 (20.0%), and we did not include incidental findings in this study. Probably it is linked to carefully selecting patients to investigate by TSO. All patients referred to TSO or TSOE panel had other leading medical problems than cancer in anamnesis or family anamnesis. In two cases, a genetic cause of disease was found using either TSO or TSOE. First, a 64-year-old man with colorectal polyposis and dentinogenesis imperfecta with a finding of NM_014208.3 (DSPP):c.52G>T (p.Val18Phe). *DSPP* gene has been previously linked to oral cancers (Joshi et al., 2010), but MMP20-DSPP co-localization and interaction has been observed in breast, colorectal and other cancers as well (Aseervatham et al., 2019). TSO/TSOE was also used to diagnose a 72-year-old woman who had had ascending colon adenocarcinoma at the age of 64 and with a family history of muscle disease; a known familial *CHEK2* variant NM_007194.3(CHEK2):c.319+2T>A p.? was confirmed in the patient, as well. The TSO panel was used to advance muscle disease diagnostics.

**TABLE 3:** Diagnostic yield of different methods used in hereditary cancer diagnostics.

Method	Total no of analyses in five years	No of analyses for CRC or polyposis in anamnesis	No of analyses with a genetic finding	Diagnostic yield of the method (%)
TruSight Cancer panel (94 genes)	2805	238	33	13.9
TruSight Cancer panel (113 genes)	899	66	8	12.1
TruSight One panel	5075	10	2	20

Altogether, around 400 MLPA analyses for different genes (*MLH1, MSH2, MSH6, PMS2, EPCAM, APC, MUTYH*) were done during the five-year study period. No new PGV or LPGV were discovered by MLPA analysis. All exon deletions were first discovered by NGS and then confirmed by MLPA.

## Discussion

Although effective diagnostic methods including NGS panels are available in Estonia, doctors still genetically test too few cases of CRC and gastrointestinal polyposis, whilst a high proportion of breast cancer cases are genetically tested. As shown in Figure 2, in the three years for which we have available data (2016–2019), we tested 2.7–6.1% of CRC (ICD-11 C18-C21) cancer patients for clinically relevant gene variants. Although testing efficacy has increased, we still have room for improvement. Testing statistics segregate more by site than by clinical problems: we have statistics of different cancer sites, but not on non-cancerous tumors and due to that these numbers are probably not 100% accurate in the context of polyposis, but still give an overview of the current situation in Estonia. In spite of the positive trend in testing over the last few years, the overall low rates of genetic testing suggest that doctors, particularly oncologists, may not know when to test for hereditary CRC and polyposis, or that they may not be aware of genetic testing resources available to them. Our study findings of a significant percentage of CRC/polyposis cases presenting with actionable variants suggest that a greater emphasis must be placed on discussing NGS testing with doctors. This should include raising awareness of the genetic underpinnings of these diseases, encouraging them to follow the international and Estonian guidelines, and collaborative case management among oncologists, oncosurgeons, gastroenterologists, family doctors etc.

In our cohort, the most used diagnostic methods were Illumina`s TruSight Cancer panels and Sanger sequencing of the familial variant in cases with known variants in a close family member. In a few cases, data reanalysis was necessary with the bigger cancer panel (TSC 113 gene panel). According to our data, there were no cases of CRC and polyposis in which pathogenic variants were identified in genes found exclusively on the 113 gene TSC panel. There was a modest difference in the diagnostic yields of the two cancer panels, likely as a result of switching to the 113 gene panel only in 2020.

Before 2019, our diagnostic workflow of CRC and polyposis consisted of TSC or TSHC together with MLPA analyses, depending on the hypothesis. This meant that the standard workflow could take up to 4–5 months for molecular diagnosis. Today, through extensive use of the CNV detection program DeCON, we can either confirm or exclude most of the hereditary cancer syndromes in only 1–2 months. From a clinical point of view, DeCON works rather efficiently, so we use different MLPA analyses primarily to confirm the alteration found, not for initial diagnosis. One important exception to this approach is identification of variants in the pseudogene, *PMS2*, for which we still need MLPA analysis to detect exon deletions. In our current clinical framework, TSO/TSOE panels are rarely used for cancer diagnostics, but we have seen that they can also provide important incidental findings in the context of hereditary cancer and polyposis syndromes.

There is still no consensus in Europe about whether it is appropriate to provide patient feedback about incidental findings on hereditary cancer syndromes. In Estonia, standard practice is to ask whether the patient or the patient`s guardian wishes to learn about findings on disease-causing alterations in the ACMG gene list. From a public health standpoint, giving feedback is cost-effective and makes the best use of available Health Insurance Funding, which covers all diagnostics used in workup, when indicated.

An American study from 2017 showed that 9.9% of CRC patients carry at least one germline pathogenic variant in a cancer susceptibility gene (Yurgelun et al., 2017). In that cohort, they found that 3.1% of CRC patients carried a pathogenic MMR gene variant, with *MLH1* being the most prevalent, while 7% carried non-Lynch syndrome gene pathogenic variants. Another study in 2021 published similar results (Uson et al., 2022). In our cohort, PGV or LPGV in MMR genes made up 37.2% of all genetic findings, of which *MLH1* LPGVs or PGVs were the most prevalent. *MLH1* mutations comprised 18.6% of all genetic findings and 50% of all MMR variants in CRC and polyposis cases in our cohort. We attribute the discordance of our findings with previously published studies to the fact that other studies were far larger, involving thousands of patients compared to our study of 314; we do not suggest that Lynch syndrome is much more prevalent among CRC patients in Estonia than in other regions.

We report a remarkable finding in our cohort of a *FANCM* disease-causing stop-gain variant NM_020937.4(FANCM):c.5791C>T in a CRC case. Until recently, *FANCM* variants had only been reported in association with breast cancer. However, a recent study beautifully confirmed that it is also familial CRC risk factor (Cannon-Albright et al., 2020). Today, we have more than one patient with this variant and indeed, it seems to segregate in CRC families. We suggest that this variant should be included in risk surveillance in association with CRC and considered when planning surveillance for healthy family members. We also report one monoallelic *MUTYH* variant in a case in which a woman had more than ten adenomatous polyps (found at 64) in her colon and had had endometrial cancer. The patient’s mother had had rectal cancer at the age of 44. *MUTYH* heterozygous variants are associated with a ∼2.5-fold increased risk of colorectal cancer compared to the general population (Win et al., 2014). Previously it had been associated with lower risk estimates (OR 1.16) in a large meta analysis (Theodoratou et al., 2010). Therefore, laboratories all over the world now report heterozygous variants to ensure early and appropriate surveillance. NCCN Guidelines version 1.2021 (www.NCCN.org) state that *MUTYH* heterozygotes should be screened by colonoscopy every 5 years from age 40, or from 10 years earlier than a first degree relative developed CRC. If there are no CRC cases in family history, the guidance is currently unclear.

Another important outstanding question is whether to report to clinicians and patients findings of variants in genes not (yet) known to be associated with CRC or polyposis, or of variants in known CRC/polyposis genes which are not yet known to be disease-causing. In an era of dramatic increases in application of NGS technologies and discoveries in disease genetics it is very important to share even variants of unknown significance through databases like ClinVar, HGMD Pro and others that help to assess the clinical relevance of variants found. Sharing this kind of data supports laboratory specialists and clinicians in reporting findings and decision making and therefor should be encouraged.

## Conclusion

In conclusion, we gathered five-year data about CRC and polyposis cases in Estonia, which were studied genetically. On the practical side, we found that the combined diagnostic yield of TruSight Cancer and TruSight Hereditary Cancer gene panels was 13.4%. We were able to optimize diagnostics using these panels in a cost-effective manner by using the program DeCON, which is specially designed for CNV calling, in *BRCA1, BRCA2, CDH1, TP53, CHEK2*, MMR genes (except *PMS2*) and *EPCAM* gene. This restricted the use of MLPA to the confirmation of diagnoses and not primary diagnoses. This shift dramatically simplified the diagnostic workflow and shortened analysis turnaround time of CRC and polyposis syndrome patients.

On the genetics side, our studies found a likely monogenic cause in 22.3% of cases overall; 21.4% of CRC cases had a likely monogenic cause, while for polyposis cases, it was 25.0%. As in previous studies, we found that the most prevalent disease-causing variants were in MMR genes, making up ∼37% of all the findings. In half of MMR cases, a pathogenic variant in the *MLH1* gene was found. These results are consistent with the fact that Lynch syndrome is the most prevalent genetic cause of colorectal cancer. Furthermore, we found five novel disease-causing gene variants, underscoring another way this work offers opportunities to share knowledge between laboratories and clinicians. Of particular interest is the FANCM NM_020937.4(FANCM):c.5791C>T variant which others have recently reported in association with CRC, which we also observe to segregate in a CRC family in the current study. This discovery highlights a broader role for FANCM as a CRC risk gene and not only as a breast cancer risk gene. Our study supports the broader use of NGS sequencing in CRC/polyposis to improve diagnosis and treatment of these conditions.

Limitations: 1) our cohort is small 2) it is a retrospective not a prospective study 3) most but not all cases are or have been tested in our laboratory, other national or abroad laboratories may have been used 4) 5 years ago mostly clinical geneticist used to order genetic testing and thus our results can be biased because of specifically choosing the group 5) the data of other characteristics is limited 6) we used diagnostic panels and definitely there are genes not covered on them for example compared to WES.

## Data Availability

The original contributions presented in the study are included in the article/supplementary materials, further inquiries can be directed to the corresponding author.

## Abbreviations

TSO, Illumina`s TruSight One panel; TSOE, Illumina`s TruSight One Expanded panel; TSC, Illumina`s TruSight Cancer panel; TSHC, Illumina`s TruSight Hereditary Cancer panel.
